# The COVID-19 Pandemic Decreases Cardiorespiratory Fitness: A 3-Year Follow-Up Study in Industry

**DOI:** 10.3390/jcdd11010009

**Published:** 2023-12-28

**Authors:** Øivind Skare, Asgeir Mamen, Marit Skogstad

**Affiliations:** 1 National Institute of Occupational Health (STAMI), Box 5330 Majorstuen, 0304 Oslo, Norway; 2 School of health Sciences, Kristiania University College, Box 1190 Sentrum, 0107 Oslo, Norway; asgeir.mamen@kristiania.no

**Keywords:** cardiorespiratory fitness, $\dot{V}$O_2max_, occupational health, COVID-19 pandemic, industry

## Abstract

Background: We aimed to determine if maximal oxygen uptake ($\dot{V}$O_2_max), resting heart rate (RHR), and self-reported leisure- time moderate to vigorous physical activity (MVPA) changed over a 3-year follow-up (FU) among industrial workers. Methods: We assessed cardiorespiratory fitness (CRF) August 2018 and August 2021. The last 17–18 months coincided with the COVID-19 pandemic. Data from 86 participants were collected; demographics by questionnaire and cardiovascular outcomes from medical examination: $\dot{V}$O_2max_, RHR, and fat mass (%). Workers reported on their leisure-time MVPA twice. To assess changes in health outcomes we applied a linear mixed model, adjusting for baseline (BL) age, sex, pack-years, shift work, and a 5-month plant shutdown. Further, we adjusted for actual age instead of BL age. Results: $\dot{V}$O_2max_ decreased from 39.6 mL/kg/min at BL to 34.0 at FU, a reduction of 5.6 mL/kg/min (95%CI, −7.6, −3.7). Adjusted for actual age, the corresponding figure for $\dot{V}$O_2max_ was 5.4 mL/kg/min, (95%CI, −7.4, −3.4), an annual loss of 4.6%. RHR increased from 61.3 to 64.4 beats per minute (95%CI, 0.8, 5.4). Self-reported MVPA decreased by 43.9 min/week, (95%CI, −73.5, −14.4). Conclusions: We observed a decrease in $\dot{V}$O_2max_, an increase in RHR and a decrease in self-reported MVPA, suggesting physical inactivity during the COVID-19 pandemic.

## 1. Introduction

The virus responsible for the COVID-19 pandemic, initially identified as a SARS-related virus, was first noticed in China in December 2019 [1]. Already in mid-March 2020, the Norwegian society shut down, with a gradual opening from May 2020 until mid-July 2020. During September 2020 and in the period December 2020-to January 2021 parts of Norway faced another round of closures; many enterprises, particularly restaurants, were closed, and the public was again encouraged to work at home. In the period January 2021 until June 2021, the number of infected decreased in line with an increase in vaccination, and by the end of September Norway was back to normal. However, in early December 2021 the Omicron-mutation was detected in Norway and new restrictions were enforced until 12 February 2022 (www.regjeringen.no, accessed on 18 February 2020). Throughout the pandemic there were temporary regional differences in closure in Norway, even within the same counties.

There has been a worldwide concern that long-lasting lockdowns due to the COVID-19 pandemic, coupled with movement restrictions, might negatively impact individuals’ health and cardiorespiratory fitness (CRF) [2]. The COVID-19 pandemic has affected physical activity and outdoor leisure activities. Although the lockdown may have increased interest in exercise, it is possible that closure of fitness facilities and prohibiting outdoor physical activity in groups such as team sports could have serious impact on public health [3].

On a general basis, literature suggests that lack of physical activity is detrimental to physical health and affects mortality [4], and that a low level of physical activity and more sedentary behavior increases the risk for chronic diseases [5]. CRF is defined as the “capacity of the circulatory and respiratory systems to supply oxygen to skeletal muscle mitochondria for energy production needed during physical activity” [6]. An objective measure of CRF is maximal oxygen uptake ($\dot{V}$O_2max_), which is measured via respiratory gases and considered the gold standard for measuring CRF [6], and $\dot{V}$O_2max_ is a marker of longevity, and a low level is a strong predictor of all-cause and disease-specific mortality [7]. Even small amounts of high intensity interval training (HIIT) improve peak oxygen consumption ($\dot{V}$O_peak_) and yield health benefits [6].

In a cohort followed for three years we have reported that shift work in industry is associated with affection of the arteries and systemic inflammation, possibly increasing the risk for future cardiovascular disease [8].

The aim of this subset study was to determine whether $\dot{V}$O_2max_, resting heart rate (RHR), and self-reported leisure-time moderate to vigorous physical activity (MVPA) changed over the 3-year follow-up (FU) in the same cohort when there were restrictions on outdoor movement and fitness centers were closed due to the COVID-19 pandemic.

We hypothesized that reduced leisure- time MVPA, stemming from these restrictions, would detrimentally affect $\dot{V}$O_2max_, RHR, and self-reported MVPA among industrial workers.

## 2. Materials and Methods

### 2.1. Study Design and Population

The present study is a prospective FU study of early manifestation of cardiovascular disease in industry in which both shift workers and day workers (controls) participate. In the period April until June 2018, we invited 172 workers, at two insulation material plants in Eastern Norway [9], whereof 94 workers agreed to participate. We examined these workers in August 2018, August 2021 and in a subset study in October 2018 [8,10]. This paper reports on the results from workers who attended at baseline (BL) and at the 3-year FU. According to the protocol, we excluded persons with serious medical conditions [9], leaving 86 eligible participants of whom we examined 70 at FU. A more detailed overview of the study design is provided elsewhere [9]. A flow diagram is provided in a former paper [8].

### 2.2. Anthropometrics

We measured body weight to the nearest 0.1 kg using a Seca 22089 scale (Seca GmbH, Hamburg, Germany) at both BL and FU. Body composition, percentage of fat mass was assessed at both occasions using the electronic weight, Tanita TBF300^®^ (Tanita Co., Tokyo, Japan), in accordance with the guidelines of the manufacturer. The subjects stood lightly clothed and barefooted on the scale until the body fat reading emerged. There was no standardization for skin temperature, hydration status or bladder volume. At BL, the participants provided their height in cm. In a questionnaire the participants reported moderate to vigorous physical activity (MVPA) in minutes per week by answering the question “How many minutes per week do you exercise on average (so that you sweat and increase your heart rate) such as jogging, cycling, spinning, swimming, playing tennis. Please state the number in minutes” [10].

As to clinical quantification of cigarette smoking, we calculated pack-years. A consumption of 20 cigarettes per day during one year is quantified as one pack-year. The number of pack years was provided for all nine daily smokers and that of the 39 former smokers (who had quit smoking one year or more prior to BL-registration), Table 1. Among the nine daily smokers only two reported that their consumption of cigarettes had changed during FU, thus the median number of cigarettes was 15 (range: 2–30) in 2018 and 15 (range: 2–20) in 2021.

### 2.3. Maximal Oxygen Uptake ($\dot{V}O_{2\max}$)

We assessed exercise capacity by a standardized graded ergometer test with a Monark 874E, ergometer cycle (Monark Exercise AB, Vansbro, Sweden) [11]. The participants started with an initial load of 70 Watts and a cadence of 70 ± 2 revolutions per minute (RPM). Thereafter, the load increased by 28 Watts (0.4 kg at 70 RPM) every minute by adding weights to the basket, with the cadence remaining constant throughout the test. When the participants failed to keep up with a cadence of minimum 60 RPM, despite encouragement, they were considered exhausted. We measured $\dot{V}$O_2max_ using a Cosmed K5 metabolism analyzer (Cosmed Srl, Rome, Italy) using the unit’s micro mixing chamber. $\dot{V}$O_2max_ was defined as the highest 30 s-averaged interval at the end of the test.

### 2.4. Resting Heart Rate (RHR)

After five minutes of rest, while sitting, we measured RHR [8]. We performed the measurements on the left arm three times in intervals of one minute. In the statistical analysis, we used the mean of three measurements. The measurements were assessed by the means of BpTRU^®^ (Bp TRU medical devices, Coquitlam, BC, Canada).

### 2.5. Statistical Analysis

Linear mixed models were used to analyze yearly change in $\dot{V}$O_2max_, s$\dot{V}$O_2_, fat mass, RHR, BMI and MVPA over the 3-years FU period. All analyses were adjusted for sex, pack-years, shift group (day workers or shift workers) and a 5-month break at one plant. For $\dot{V}$O_2max_, fat mass, RHR and BMI we also adjusted for BL MVPA. Furthermore, in all analyses we adjusted for age. In one set of analyses, we adjusted for BL age, while in the other we adjusted for actual age at the time of measurements. All analyses were done in Stata (StataCorp, 2023. *Stata Statistical Software: Release 18*. StataCorp LLC, College Station, TX, USA). *p*-values below 0.05 were considered significant.

## 3. Results

### 3.1. Demographic Characteristics of the Study Population

There was no difference in body mass index (BMI) when BL values were compared with those of FU. Median fat mass did not change during FU, Table 2. At FU, the participants reported a lower degree of MVPA than what they reported at BL, Table 2.

### 3.2. Maximal Oxygen Uptake ($\dot{V}O_{2\max}$)

Seventy-three participants performed $\dot{V}$O_2max_ at BL, 33 at FU. Those who participated in the $\dot{V}$O_2max_ test at FU were not significantly different from those who did not when it comes to reported PA, measured BMI, and $\dot{V}$O_2max_ at BL (results not shown). The $\dot{V}$O_2max_ decreased significantly from 39.5 mL/kg/min at BL to 34.1 at FU, a difference of −5.4 mL/kg/min (95%CI, −7.4, −3.4), when adjusting for actual age. The annual decrease was estimated to be 4.6%. A significant decrease in s$\dot{V}$O_2_ was also noted, Table 2. Upon examining the correlation between $\dot{V}$O_2max_ measurements at BL and after two months, we observed a correlation coefficient of 0.864. Between the baseline and the three-year follow-up, the correlation was found to be 0.830. A scatter plot is provided in the Supplementary file.

### 3.3. Resting Heart Rate (RHR)

There was a significant increase in RHR over the 3-year FU period of 61.3 to 64.4 beats per minute (95%CI, 0.8, 5.4), Table 2.

## 4. Discussion

This 3-year follow-up (FU) study of industrial workers reveals a decrease in $\dot{V}$O_2max_ that exceeds what would be expected due to aging. Additionally, RHR increased and self-reported leisure-time MVPA decreased during a period that overlapped with 17–18 months of the COVID-19 pandemic.

At BL, $\dot{V}$O_2max_ values were within what is considered normal for a comparable Norwegian healthy population [12] or even exceeding that of other Europeans [13]. In the present population, the annual reduction in $\dot{V}$O_2max_ was 4.6%, which is not solely attributable to aging, as the general decline in $\dot{V}$O_2max_, suggests an annual loss of only 0.5–1% [14,15]. The dramatic reduction in $\dot{V}$O_2max_ is indicative of decreased physical activity, since reductions in training volume seems to positively correlate with decline in $\dot{V}$O_2max_ [16].

We observed an increase in RHR during the 3-year FU. Increased RHR can result in increased blood pressure (BP), dyslipidemia and obesity, insulin resistance, all in all increasing the risk for atherosclerosis [17]. This might explain why individuals with high RHR have an increased risk for cardiovascular disease (CVD) compared to those with lower or stable RHR [18,19]. During the 3-year FU we noted an increase of 3 beats per minute (bpm). This suggests an elevated risk for CVD since a 5-bpm increase is associated with an 11.3% higher relative risk for fatal and non-fatal myocardial infarction in clinical settings [20]. Low RHR, on the other hand, is beneficial for the heart, reflecting less hemodynamic and sympathetic stress and diminished mechanical load. Intense training also decreases arterial stiffness and thus the strain on myocardium [18,19,21].

Self-reported MVPA levels decreased during FU in our study. A decrease in physical activity could affect energy expenditure and potentially increase CVD risk factors, including increased weight. However, BMI and fat mass remained consistent over the three years of follow-up. This stability in BMI and fat mass does not, however, negate the possibility of adverse effects on the fat metabolism. A reduction in energy expenditure can lead to increased intra-abdominal fat without a corresponding rise in BMI [22]. Such an increase in obesity is associated with increased inflammation, disturbance of lipid metabolism, increased blood pressure, decreased insulin sensitivity and, consequently, a higher risk for diabetes 2, and CVD events [6,22,23].

Throughout the study, workers continued their regular duties at the plant. This suggests that their daily physical activity remained relatively consistent from BL to FU. Physical activity in an occupational setting, however, does not appear to offer the same protective benefits against CVD as leisure-time activity [24] which, in our study, was reported to decrease during FU. Therefore, the reasons for our observed reduction in self-reported MVPA during FU likely lie in the external circumstances faced by workers during FU.

The latter part of the FU, spanning 17–18 months, coincided with the COVID-19 pandemic. Initially, there were some restrictions of movement outdoors in Norway. Later, fitness centers remained closed for many months and organized outdoor physical activity in groups was prohibited. Compared to fitness centers in the local community, the ones in the plants were blocked for even longer periods; workers had restricted or no access from March 2020 to March 2022 (Xander Nordli-Bergsholm, personal communication).

The COVID-19 pandemic has negatively impacted physical activity [25,26], and a shift towards sedentary behavior is known to dramatically reduce $\dot{V}$O_2max_ [27]. It is plausible that the result of the present study is a consequence of lack of leisure-time MVPA during the COVID-19 pandemic. A significant decline in physical fitness due to the COVID-19 pandemic has also been reported among children and adolescents [28].

A strength of the present study is its prospective design with information on MVPA and examination of $\dot{V}$O_2max_ and RHR during FU. Using the same technicians throughout the study period ensured consistency, and measurements were taken at the same time of the day to avoid diurnal variation. In addition to BL and FU, we measured $\dot{V}$O_2max_ in November 2018 and there were minimal differences when these results were compared to those at BL [10]. Therefore, it is likely that the reduction in $\dot{V}$O_2max_ during the FU is valid and not a result of methodological or technical challenges.

A limitation of the study is the relatively small number of workers performing the $\dot{V}$O_2max_ test at FU. However, regarding BL-measures, these workers did not differ significantly from the group of workers who performed the test. The group of workers in the present study may not represent the general Norwegian population. However, $\dot{V}$O_2max_ at BL was in line with what is considered normal for a corresponding general population sample [12]. Furthermore, leisure-time MVPA was not measured objectively, which is a limitation since self-reported physical activity can introduce response bias or even misclassification and seems to be overestimated [29]. However, it seems that self-reported vigorous physical activity is highly correlated with $\dot{V}$O_2max_ in a population sample of men in Norway [30]. There was a lack of registration of low activity. However, we believe that this type of activity remained consistent from BL to FU since the workers were working during the entire FU period. Lastly, at FU the participants were asked if they had any disease, but we did not register long-covid specifically at FU nor did we register the number of participants who had been ill with COVID-19 during the 3-year follow-up.

## 5. Conclusions

The findings from this 3-year FU study, including decreased $\dot{V}$O_2max,_ increased RHR and reduced self-reported leisure-time MVPA, suggest that diminished leisure-time MVPA during the COVID-19 pandemic has impacted industrial workers’ cardiorespiratory fitness, potentially indicating an increased risk for future CVD.

## Figures and Tables

**Table 1:** Baseline measurements of the 86 workers participating in the study.

Variables	No	Min	Max	Mean	SD
Age (years)		21.0	62.0	42.0	11.2
Women (number)	12 (14%)				
Shift workers (number)	57 (66%)				
Participants with a 5-month break (number)	12 (14%)				
BMI (kg/m^2^)		18.9	39.7	27.0	4.6
Fat mass (%) ^a^		9.8	44.9	26.0	7.8
MVPA (min/week) ^b^		0.0	700.0	95.6	126.0
HF max		149.0	209.0	178.6	12.6
Smokers (number)	9 (10%)				
Pack-years ^c^		0.0	43.0	7.0	9.7

^a^ No = 82; ^b^ No = 85; ^c^ No = 39 former and nine daily smokers; No, Number of participants; BMI, body mass index; MVPA, moderate to vigorous physical activity, self-reported; HF max, maximum heart rate; pack-years, clinical quantification of cigarette smoking.

**Table 2:** Results from a 3-year follow-up of 86 industrial workers in the period August 2018–August 2021.

	Adjustment Includes BL Age						Adjustment Includes Actual Age					
Outcome	Mean BL	Mean FU	Change	Lower	Upper	*p*-Value	Mean BL	Mean FU	Change	Lower	Upper	*p*-Value
$\dot{V}$O_2max_ (mL/kg/min) *	39.6	34.0	−5.6	−7.6	−3.7	<0.001	39.5	34.1	−5.4	−7.4	−3.4	<0.001
s$\dot{V}$O_2_ (mL/kg^0.67^/min)	170.2	146.2	−24.0	−52.6	−9.3	<0.001	169.9	146.9	−23	−48.2	−3.2	<0.001
Fat Mass (%) *	26.2	26.2	0.0	−1.2	1.2	1.0	26.3	26.0	−0.3	−1.5	1.0	0.67
RHR (beats/min) *	61.3	64.4	3.1	0.8	5.4	0.0077	61.0	64.7	3.7	1.4	6.1	<0.002
BMI (kg/m^2^) *	27.1	27.5	0.4	0.0	0.8	0.031	27.1	27.4	0.3	−0.1	0.8	0.16
MVPA (min/week) **	103.8	59.9	−43.9	−73.5	−14.4	0.0036	100.5	64.0	−36.4	−66.5	−6.4	0.017

BL, BL in 2018; FU, FU in 2021; $\dot{V}$O_2max_, the maximum rate of oxygen consumption attainable during physical exertion reflecting cardiorespiratory fitness; s$\dot{V}$O_2_, oxygen uptake allometrically scaled (BM raised to the power of 2/3); RHR, Resting heart rate; BMI, body mass index; MVPA, moderate to vigorous physical activity, self-reported. * Also adjusted for sex, pack-years, shift group, five months break at a plant and physical activity. ** Also adjusted for sex, pack-years, shift group and a 5-months break at a plant.

## Data Availability

Data are contained within the article and supplementary materials.

## Supplementary Materials

The following supporting information can be downloaded at: https://www.mdpi.com/article/10.3390/jcdd11010009/s1.
