# Supplementary material for: The COVID-19 Pandemic Decreases Cardiorespiratory Fitness: A 3-Year Follow-Up Study in Industry
Source: J Cardiovasc Dev Dis. 2023 Dec 28;11(1):9. doi: 10.3390/jcdd11010009 (PMC10816953; doi:10.3390/jcdd11010009)

## Supplementary Figure S1

Upon examining the correlation between $VO_2$max measurements at baseline (BL) and after two months, we observed a correlation coefficient of 0.864 ($p<0.001$). Between the baseline and the three-year follow-up, the correlation was 0.830 ($p<0.001$). Presented here is a scatter plot showing $VO_2$max values at baseline and at the three-year follow-up.

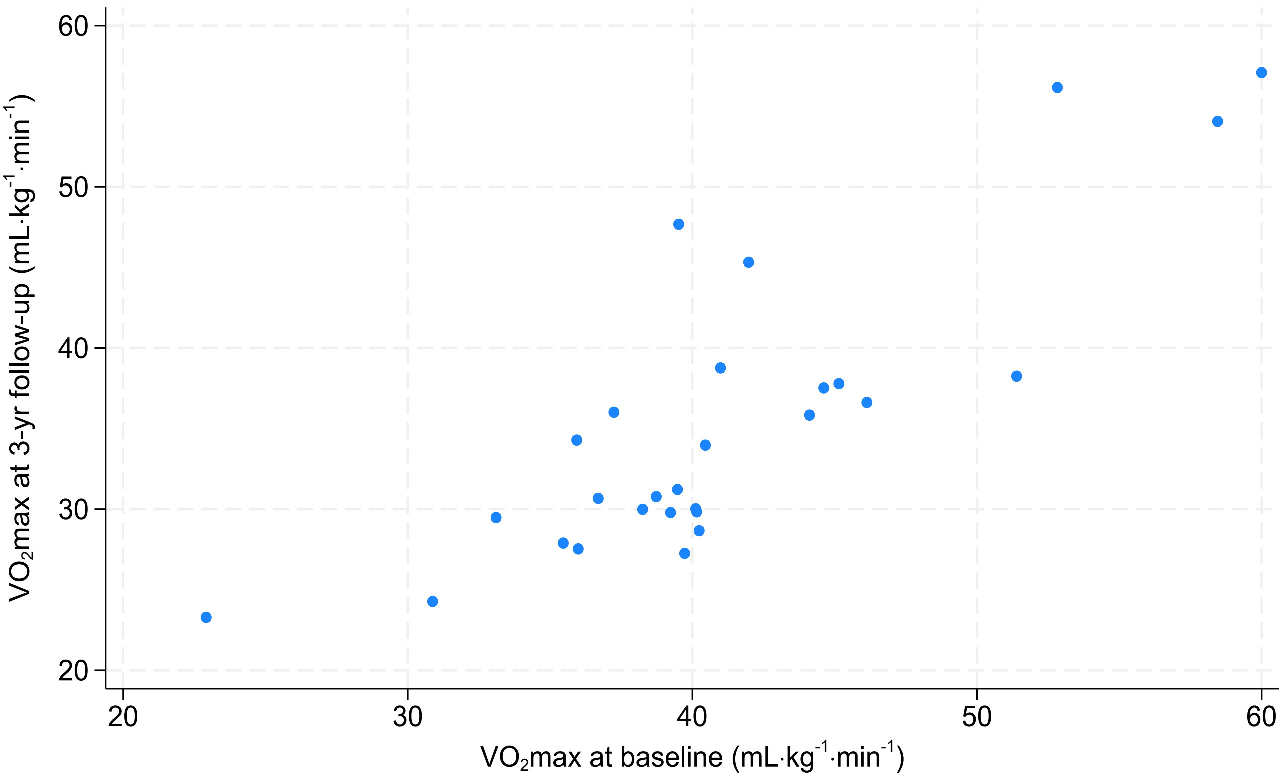

Supplement: Supplementary file 1 [file jcdd-11-00009-s001.zip › supplementary.pdf]
